# Comparative Analysis of the Complete Chloroplast Genomes in *Allium* Subgenus *Cyathophora* (Amaryllidaceae): Phylogenetic Relationship and Adaptive Evolution

**DOI:** 10.1155/2020/1732586

**Published:** 2020-01-17

**Authors:** Xin Yang, Deng-Feng Xie, Jun-Pei Chen, Song-Dong Zhou, Yan Yu, Xing-Jin He

**Affiliations:** Key Laboratory of Bio-Resources and Eco-Environment of Ministry of Education, College of Life Sciences, Sichuan University, Chengdu, China

## Abstract

Recent advances in molecular phylogenetics provide us with information of *Allium* L. taxonomy and evolution, such as the subgenus *Cyathophora*, which is monophyletic and contains five species. However, previous studies detected distinct incongruence between the nrDNA and cpDNA phylogenies, and the interspecies relationships of this subgenus need to be furtherly resolved. In our study, we newly assembled the whole chloroplast genome of four species in subgenus *Cyathophora* and two allied *Allium* species. The complete cp genomes were found to possess a quadripartite structure, and the genome size ranged from 152,913 to 154,174 bp. Among these cp genomes, there were subtle differences in the gene order, gene content, and GC content. Seven hotspot regions (*infA*, *rps16*, *rps15*, *ndhF*, *trnG-UCC*, *trnC-GCA*, and *trnK-UUU*) with nucleotide diversity greater than 0.02 were discovered. The selection analysis showed that some genes have elevated Ka/Ks ratios. Phylogenetic analysis depended on the complete chloroplast genome (CCG), and the intergenic spacer regions (IGS) and coding DNA sequences (CDS) showed same topologies with high support, which revealed that subgenus *Cyathophora* was a monophyletic group, containing four species, and *A. cyathophorum* var. *farreri* was sister to *A. spicatum* with 100% bootstrap value. Our study revealed selective pressure may exert effect on several genes of the six *Allium* species, which may be useful for them to adapt to their specific living environment. We have well resolved the phylogenetic relationship of species in the subgenus *Cyathophora*, which will contribute to future evolutionary studies or phylogeographic analysis of *Allium*.

## 1. Introduction

**Figure 1:**
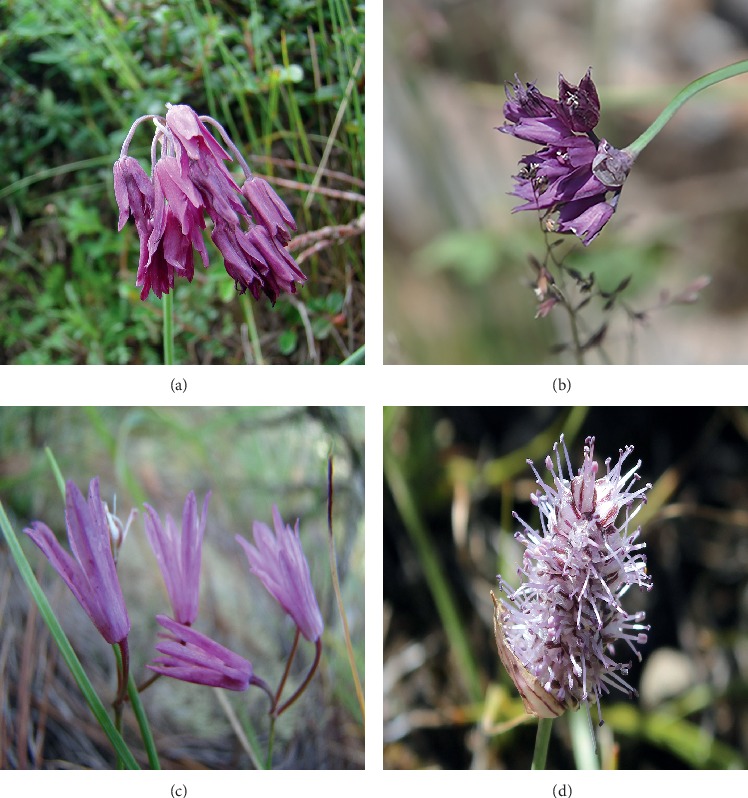
The morphological characters of flowers of subgenus *Cyathophora* species. (a) *A. cyathophorum*. (b) *A. cyathophorum* var. *farreri*. (c) *A. maire*i. (d) *A. spicatum*.

Subgenus *Cyathophora* (R. M. Fritsch) R. M. Fritsch is a small group of *Allium* that has been put forward lately [1]. The special subgenus *Cyathophora* contains about six species and one variety according to Li et al. [2]; besides, *A. spicatum* (Prain) N. Friesen has a wild distribution range, extending from China to Nepal, while the rest of them are endemic species in China and mainly distributed in the southeastern margin of the Qinghai-Tibet Plateau (QTP): *A. mairei* Lév, *A. kingdonii* Stearn, *A. rhynchogynum* Diels, *A. trifurcatum* (F. T. Wang and Tang) J. M. Xu, and *A. cyathophorum* Bur. and Franch and its variety *A. cyathophorum* var. *farreri* (Stearn) Stearn. Although it contains a small number of species, the boundary of subgenus *Cyathophora* and the involved species have experienced some alterations with the development of molecular biology. In previous study, *A. spicatum* was classified at different taxonomic levels because of its idiographic spicate inflorescence based on morphological and molecular evidences [3]. Five species have been proposed by Huang et al. about subgenus *Cyathophora* [4]: *A. mairei*, *A. rhynchogynum*, *A. cyathophorum*, *A. cyathophorum* var. *farreri*, and *A. spicatum*, while *A. kingdonii* and *A. trifurcatum* did not belong to this group. Micromorphological and cytological features supported that the subgenus *Cyathophora* is a monophyly and contains five species [5, 6]. Among species of the subgenus *Cyathophora*, *A. spicatum* grows in the droughty western QTP with the extremely abnormal spicate inflorescence [3], while *A. cyathophorum* and *A. cyathophorum* var. *farreri* with the umbel inflorescence stretch to the moist HMR [7] (Figure 1). Furthermore, Li et al. [6] suggested that *A. cyathophorum* and *A. farreri* were independent species based on molecular phylogeny and the striking distinctiveness in micromorphology. *A. rhynchogynum* has never been sampled since it was published in 1912 [8]. We also performed a lot of field work to collect it but failed. The Flora of China recorded that *A. rhynchogynum* only distributed in northwest of Yunnan province in China. Therefore, we speculate that *A. rhynchogynum* might become extinct or there is an identification error in previous research studies. Li et al. [6] performed phylogenetic and biogeographic analyses for *A*. *cyathophorum* and *A*. *spicatum* based on chloroplast and nuclear ribosomal DNA and detected distinct different topologies between these two molecular methods, in which *A. cyathophorum* showed close relationship with *A. spicatum* in nuclear DNA tree but was sister to *A. cyathophorum* var. farreri in cpDNA tree [4, 6]. Other than this, the relationship between these species is not exactly determined, and phylogenetic analysis using single or several combined chloroplast fragments does not solve the problem effectively, and the complete cp genome can well resolve the relationship of subgenus *Cyathophora*. Hence, it is imperative to reconstruct the relationship of subgenus *Cyathophora* and clarify the contained species depending on the complete chloroplast genomes. To evaluate the subgenus *Cyathophora* resources comprehensively, we also need more efficient molecular markers.

Chloroplast is one of the basic organelles in plant cells, which is in charge of photosynthesis of green plants [9]. The chloroplast genomes have a highly conserved structure and gene content, which have a quadripartite structure composed by large single-copy (LSC) and small single-copy (SSC) regions separated by two parts of inverted repeat (IR) [10, 11]. Previous studies suggested that genome size of angiosperms ranged from 120 kb to 170 kb with gene number changed from 120 to 130 [12]. Complete chloroplast genome has long been a core issue in plant molecular evolution and systematic studies because of its oversimplified structure, highly conservative sequence, and maternal hereditary traits [13]. Since the complete cp genome analysis can provide more genetic information contrasted with just single or few cpDNA fragments [14], by using cp genome sequences, many long existing phylogenetic problems of different angiosperms at various taxonomic levels have been successfully resolved [15–20].

In addition to exploring phylogenetic studies, the whole cp genome has important significance to reveal the photosynthesis mechanism, metabolic regulation, and adaptive evolution of plants. Research has shown that adaptive evolution is mainly promoted by evolutionary processes like natural selection, which affects genetic changes caused by genetic recombination and mutations [21]. Many recent studies have analyzed the selection pressures that undergo by species in the evolutionary processes based on complete chloroplast genome, for example, a positive selection for the *atpF* gene may suggest that it has made an important impact on the divergence in deciduous and evergreen oak tree [9], and there also existed positive selection on *ycf2* in watercress chloroplasts [22]. With the development of sequencing technology, the number of cp genomic sequences has increased dramatically in recent years. However, a few plastid genomes of *Allium* were reported until now, and it is necessary to develop more complete chloroplast genome in *Allium* for future phylogenetic and evolutionary research studies.

In our report, we assembled and characterized the complete cp genome sequence of the six *Allium* species using next-generation sequencing technologies to (1) reveal common structural patterns and hotspot regions, (2) gain a better understanding of the relationship about subgenus *Cyathophora* based on complete chloroplast genome, and (3) investigate adaptive evolution in the cp genomes of the six *Allium* species. We hope our study will provide valuable genetic resources for further evolutionary studies about subgenus *Cyathophora*.

## 2. Materials and Methods

### 2.1. Plant Materials, DNA Extraction, and Sequencing

Fresh leaves of *A. cyathophorum*, *A. cyathophorum* var. *farreri*, *A. spicatum*, *A. maire*i, *A. trifurcatum*, and *A. kingdonii* were collected from different places (Table 1). Morphological characters were measured using karyotype [23]. The healthy leaves were immediately dried with silica gel to use for DNA extraction. The voucher specimens were stored in the Herbarium of Sichuan University (SZ Herbarium). Their total genomic DNA was extracted from the sampled leaves according to the manufacturer's instructions for the Plant Genomic DNA Kit (Tiangen Biotech, Beijing, China). Genomic DNA was indexed by tags and pooled together in one lane of Illumina HiSeq platform for sequencing (paired-end, 350 bp) at Novogene (Beijing, China).

### 2.2. Chloroplast Genome Sequence Assembly and Annotation

We firstly used FastQC v0.11.7 to assess the quality of all reads [24]. To select the best reference, we filtrated the chloroplast genome related reads by mapping all reads to the published chloroplast genome sequences in *Allium*. SOAPdenovo2 was used to assemble all relevant reads into contigs [25]. The clean reads were assembled using the program NOVOPlasty [26] with the complete chloroplast genome of its close relative *A. cepa* as the reference (GenBank accession no. KM088014). Geneious 11.0.4 was used to finish the annotation of the assembled chloroplast genome, and it was corrected manually after comparison with references [27]. The circular plastid genome maps were generated utilizing the OGDRAW program [28]. The GenBank accession numbers of *A. cyathophorum*, *A. cyathophorum* var. *farreri*, *A. spicatum*, *A. mairei*, *A. trifurcatum*, and *A. kingdonii* are MK820611, MK931245, MK931246, MK820615, MK931247, and MK294559, respectively.

### 2.3. Repeat Sequences and Simple Sequence Repeat (SSR) Analysis

REPuter [29] was selected to investigate the location and size of repeat sequences, which included four types of repeats in the chloroplast genomes about the six *Allium* species. The sequence identity and minimum length of repeat size was set to >90% and 30 bp, with the hamming distance of 3. We used IMEx, ImperfectMicrosatelliteExtractor (http://43.227.129.132:8008/IMEX/imex_advanced.html) [30], to find chloroplast SSRs in six chloroplast genome sequences of *Allium*. Its specifications were set up as follows: the minimum number of repeats for mononucleotide, dinucleotides, trinucleotides, tetranucleotides, pentanucleotide and hexanucleotides was 10, 5, 5, 4, 3, and 3, respectively, the repeat type was imperfect, the imperfection % was Mono: 10%, Di: 10%, Tri: 15%, Tetra: 20%, Penta: 5%, and Hexa: 5%, mismatches allowed in pattern were Mono: 1, Di: 1, Tri: 2, Tetra: 4, Penta: 0, and Hexa: 3, and the level of standardization was level 1 standardization.

### 2.4. Codon Usage Analysis

Codon usage of the species in subgenus *Cyathophora* was analyzed by the software of CodonW [31]. Protein-coding genes (CDS) were selected with the following filter requirements: (1) each CDS was longer than 300 nucleotides [18, 32]; (2) repeat sequences were deleted. Totally, 53 CDS of each species in *Allium* were selected for further study.

### 2.5. Genome Comparison (IR Contraction and Expansion)

The mVISTA program was chosen to analyze the whole sequence similarity of all six *Allium* species with Shuffle-LAGAN model [33], using the chloroplast genomes to compare their difference in sequences at the chloroplast genome level and *A. cyathophorum* as the reference. The boundaries between single copy regions (LSC and SSC) and inverted repeats (IR) regions among the six chloroplast genome sequences were compared by using Geneious v11.0.4 software [27].

### 2.6. Hotspot Regions Identification in Subgenus *Cyathophora*

To analyze nucleotide diversity (Pi), we extracted the shared 112 genes of the six species in *Allium* after alignment. DnaSP 5.10 was employed to calculate the nucleotide variability [34].

### 2.7. Gene Selective Pressure Analysis of Six *Allium* Plastomes

To investigate selection pressures, nonsynonymous (Ka) and synonymous (Ks) substitution rates of 65 selected protein-coding genes between the cp genomes of subgenus *Cyathophora* and the other two *Allium* species were calculated by KaKs Calculator version 2.0 [35].

### 2.8. Subgenus *Cyathophora* Phylogenomic Analysis Based on Chloroplast Genome

Phylogenetic analysis of subgenus *Cyathophora* was totally depended on twenty-nine complete chloroplast genome sequences, which were twenty-one species of *Allium* (including 6 newly assembled species; 15 other species of *Allium* were collected from NCBI), six species of *Lilium*, and two species of *Asparagus* as the out groups (Table S1). Three different databases were used to build the phylogenetic tree, which include the complete genome sequences, the IGS sequences, and all CDS sequences, and three different methods, Bayesian-inference (MrBayes v3.2), maximum parsimony (PAUP-version4.0), and maximum likelihood (RAxmL8.0), were used to build the tree. The sequences were aligned using MAFFT [36] in Geneious 11.0.4 with the set parameters and manually trimmed. GTR + I + G was selected as the best model using software ModelTest v3.7 [37]. Maximum likelihood (ML) analyses were performed using RAxmL8.0 with 1000 bootstrap replications [38]. PAUP was used to conduct maximum parsimony (MP) analyses [39]. MP was run using a heuristic search with 1000 random addition sequence replicates with the tree-bisection-reconnection (TBR) branch-swapping tree search criterion. Bayesian inference (BI) was executed with Mrbayes v3.2 [40], and the Markov chain Monte Carlo (MCMC) analysis was run 1 × 10^8^ generations. The trees were sampled every 1000 generations: the first 25% were discarded as burn in and the remaining trees were used to establish a 50% majority rule consensus tree. When the average standard deviation of the splitting frequency was kept below 0.001, it was considered that the stationarity is achieved.

## 3. Results

### 3.1. Chloroplast Genome Organization and Gene Content in Six Species

**Figure 2:**
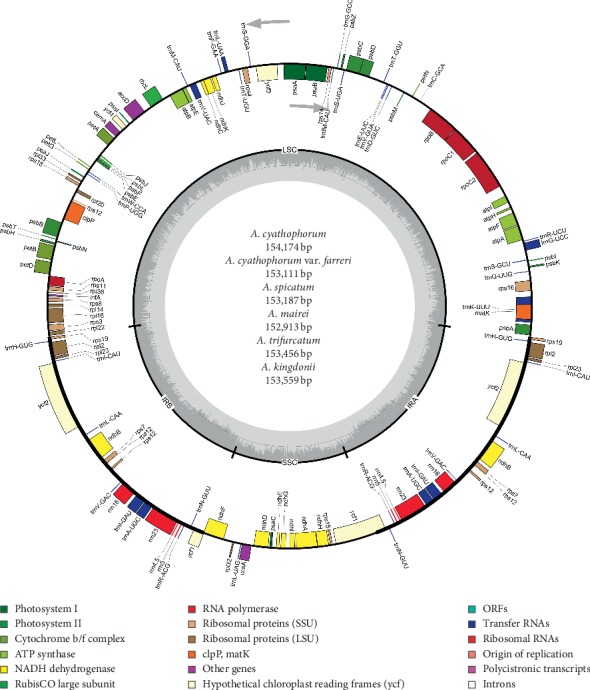
Gene map of *A. cyathophorum*, *A. cyathophorum* var. *farreri*, *A. spicatum*, *A. mairei*, *A. trifurcatum*, and *A. kingdonii* complete chloroplast genomes. The circle inside genes is transcribed clockwise, and the outside genes are transcribed counterclockwise. Genes belonging to different functional groups are represented by distinct colors.

These six acquired *Allium* cp genomes were detected to have a circular DNA structure of angiosperm cp genomes that comprises LSC, SSC, and two IR regions (Figure 2). The sizes of the six CP genomes ranged from 152,913 bp for *A. mairei* to 154,174 bp for *A. cyathophorum*, which were similar with other *Allium* CP genomes [41]. The size varied from 82,493 bp (*A. mairei*) to 83,423 bp (*A. kingdonii*) in the LSC region, from 17,811 bp (*A. kingdonii*) to 21,706 bp (*A. trifurcatum*) in the SSC region, and from 24,561 bp (*A. trifurcatum*) to 26,467 bp (*A. cyathophorum*) in the IR region (Table 2). The entire GC content of the cp genome sequences was 36.8–36.9%, and the GC contents of the LSC, SSC, and IR regions were 34.6–34.8%, 29.5–31.2%, and 42.7–43.1%, respectively. A total of 132 genes were discovered from the complete cp genome: 8 ribosomal RNA (rRNA) genes, 86 protein-coding genes, and 38 transfer RNA (tRNA) genes (Table 3).

### 3.2. Repeat and Simple Sequence Repeat (SSR) Analysis

**Figure 3:**
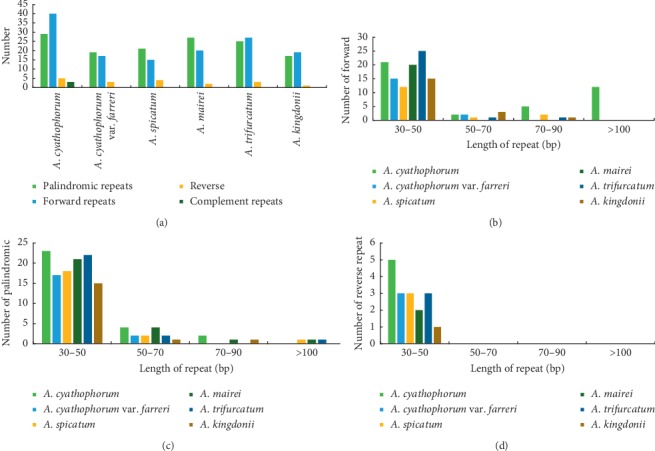
Investigation of repeated sequences in *A. cyathophorum*, *A. cyathophorum* var. *farreri*, *A. spicatum*, *A. mairei*, *A. trifurcatum*, and *A. kingdonii* chloroplast genomes. (a) Four repeat types. (b) Number of the forward repeat by length. (c) Number of the palindromic repeat by length. (d) Number of the reverse repeat by length.

Many research studies of cp genomes revealed that repeat sequences have been widely used in phylogeny, population genetics, and other studies [42]. Four types of repeats (forward repeats, reverse repeats, complement repeats, and palindromic repeats) were detected in the six *Allium* species. There were only 3 complement repeats in *A. cyathophorum*, while the other species did not have. The number of repeats varied from 37 to 77 in the six species; the *A. cyathophorum* showed the most abundant number of repeats, including 29, 40, 5 and 3 palindromic forward reverse and complement repeats, respectively. The number of forward repeats ranged from 15 to 40, the number of palindromic repeats ranged from 17 to 29, and the number of reverse repeats ranged from 1 to 5 (Figure 3). The lengths of forward, palindromic, and reverse repeats ranged from 30 to 267 bp, and most of them were concentrated in 30–50 bp (81.48%), while those of 50–70 bp (9.09%), >100 bp (6.40%), and 70–90 bp (3.03%) were less common (Figure S1). Earlier reports recommend that the appearance of the repeats indicates that this locus is a staple hots-pot for reconfiguration of the genome [43–45]. Nevertheless, these repeats are valuable for developing genetic markers in population genetics studies [46, 47].

**Figure 4:**
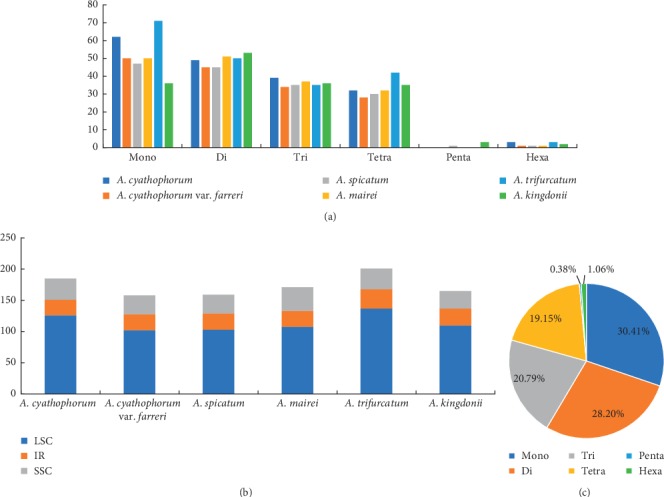
Analysis of simple sequence repeats (SSRs) in six *Allium* chloroplast genome sequences. (a) Number of six SSR types discovered in six *Allium* chloroplast genome sequences. (b) Number of SSRs in the LSC, IR, and SSC regions in six *Allium* chloroplast genome sequences. (c) Presence of different SSR types in total SSRs of six *Allium* chloroplast genome sequences.

SSRs, also called as microsatellites, are 1- to 6-bp repeating sequences that are extensively distributed in the chloroplast genome. SSRs are highly polymorphic and codominant, which are valuable markers for study involving gene flow, population genetics, and gene mapping [48]. In this study, six classes of SSRs (mono-, di-, tri-, tetra-, penta-, and hexanucleotide repeats) were found in the cp genome of the six species, whereas the number of hexanucleotide repeats ranged from 1 to 3 in the six species, and the pentanucleotide repeats just existed in *A. kingdonii* and *A. spicatum*. The total number of SSRs in the genome of the six *Allium* species was 185 in *A. cyathophorum*, 158 in *A. cyathophorum* var. *farreri*, 159 in *A. spicatum*, 171 in *A. mairei*, 201 in *A. trifurcatum*, and 165 in *A. kingdonii* (Figure 4(a)). The highest number was mononucleotide repeat, which accounted for about 30.41% of the total SSRs (Figure 4(c)); the number ranged from 36 in *A. kingdonii* to 71 in *A. trifurcatum*, and all mononucleotide repeats are composed of A or T bases; these conclusions were unanimous in previous studies that SSRs in cp genomes usually contained short polyA or polyT repeats [49], while those of dinucleotide repeats (28.20%), trinucleotide repeats (20.79%), tetranucleotide repeats (19.15%), pentanucleotide repeats (0.38%), and hexanucleotide repeats were the least abundant (1.06%). In the whole SSR locus, the SSRs located in the LSC area are much more than those in the SSC and IR areas (Figure 4(b)), which is identical with previous research studies that SSRs are unevenly distributed in cp genomes [50].

### 3.3. Codon Usage Analysis

Codon usage bias is a phenomenon that the synonymous codons usually have different frequencies of use in plant genomes, which was caused by evolutionary factors that affect gene mutations and selections [51, 52]. The relative synonymous codon usage (RSCU) is a method that estimates nonuniform synonymous codon usage in coding sequences, in which RSCU less than 1 demonstrates lack of bias, whereas RSCU value greater than 1 stands for more frequent use of a codon. In view of the sequences of 53 protein-coding genes (CDS), the codon usage frequency was calculated for the six *Allium* cp genomes (Table 4). Altogether, the number of codons ranged from 33058 in *A. kingdonii* to 23791 in *A*. *mairei*. In addition, the result indicated that a total of 13218 codons encoding leucine in the cp genomes of the six species and 1453 codons encoding cysteine as the most common and least common universal amino acids, respectively. As recently discovered in other cp genomes of plants, our study revealed that except tryptophan and methionine, there was preference in the use of synonymous codons, and the RSCU value of 30 codons exceeded 1 for each species, and they were A or T-ending codons. The result is in accordance with other researches, which the codon usage preference for A/T ending in plants [53–55].

### 3.4. Comparative Analysis of the Chloroplast Genomes among Six Species in *Allium*

**Figure 5:**
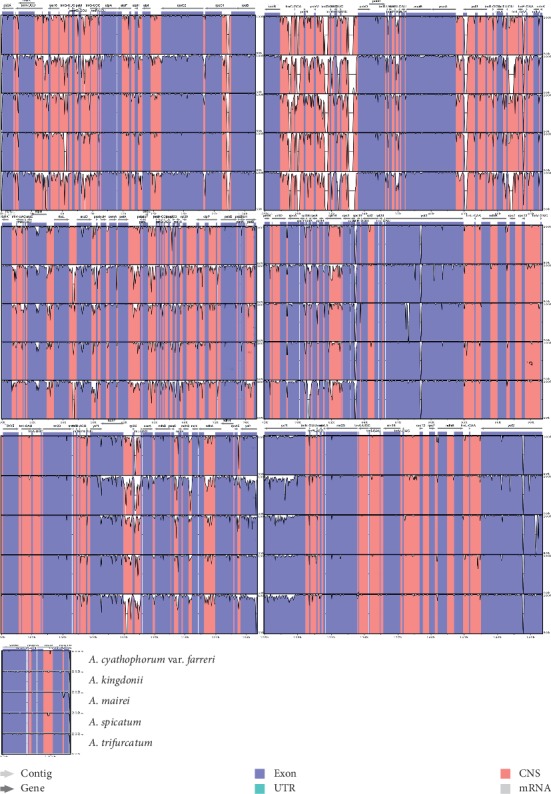
Sequence alignment of six *Allium* chloroplast genomes (*A. cyathophorum* as the reference). The *y*-axis represents the percent similarity between 50% and 100%. Different colors represent different genetic regions.

mVISTA online software in the Shuffle-LAGAN mode was employed to analyze the comprehensive sequence discrepancy of the six chloroplast genomes of *Allium* with the annotation of *A. cyathophorum* as a reference. In this study, the whole chloroplast genome alignment showed great sequence consistency of the six cp genomes, indicating that *Allium* cp genomes are very conservative (Figure 5). We found that among the six cp genomes, their IR region is more conserved compared to the LSC and SSC regions, which is similar with other plants [56, 57]. Furthermore, as we have found in other angiosperms, the coding areas were more conserved than the noncoding areas, and there were more variations in the intergenic spacers of the LSC and SSC areas, whereas the IR areas presented a lower sequence divergence [58, 59]. *A. cyathophorum* var. *farreri* had the highest sequence similarity to *A. cyathophorum* in sequence identity analysis. Noncoding regions displayed varying degrees of sequence differences in these six *Allium* cp genomes, including *trnK-rps16*, *trnS-trnG*, *atpH-atpI*, *petN-psbM*, *trnT-psbD*, *trnF-ndhJ*, *accD-psaI*, and *petA-psbL*. The coding areas with significant diversity contain *matK*, *rps16*, *rpoC2*, *infA*, *ycf1*, *ndhF*, and *rps15* genes. The highly diverse regions found in this study may be used to develop molecular markers that can improve efficiency to study phylogenetic relationships within the *Allium* species.

**Figure 6:**
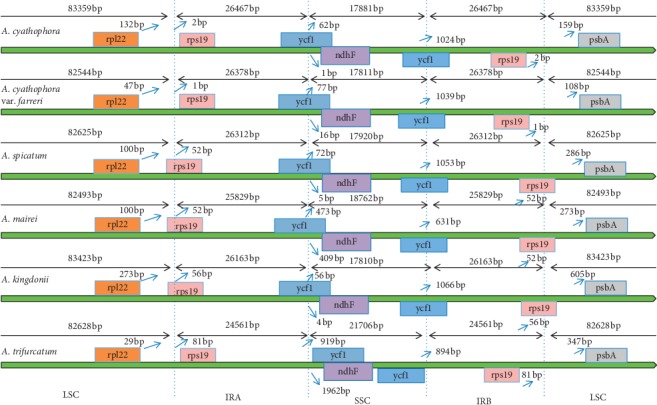
Comparison of the boundaries of the LSC, SSC, and IR areas of the whole chloroplast genomes of the six species.

Though the cp genome is usually well conserved, having typical quadripartite structure, gene number, and order, a phenomenon recognized as ebb and flow exists, and this is where the IR area often expands or contracts [60]. Expansion and contraction of IR region is related to the size variations in the cp genome and has great differences in its evolution [61, 62]. We compared the IR/SC boundary areas of the six *Allium* cp genomes, and we found that there are obvious differences in the IR/LSC and IR/SSC connections (Figure 6). At the boundary of LSC/IRa junction, *rps19* gene of different species distance the boundary were from 1 to 81 bp, while the *rpl22* genes distance the border were from 29 to 273 bp. At the boundary of LSC/IRb connections, the *psbA* genes distance the border were reached from 108 to 605 bp. The inverted repeat b (IRb)/SSC border located in the coding region, and the *ycf1* genes of the six species with a region ranged from 4193 to 5223 bp located in the SSC regions, which the *ycf1* gene of *A. trifurcatum* all located in the SSC region. The shorter *ycf1* gene crossed the inverted repeat (IRa)/SSC boundary, with 56–919 bp locating in the SSC regions. And the *ndhF* genes were situated in the SSC regions, which distance from the IRa/SSC boundary ranged from 1 to 1962 bp. Undoubtedly, the full-length differences in the sequence of the six cp genomes are caused by changes in the IR/SC boundaries.

### 3.5. Hotspot Regions Identification in Subgenus *Cyathophora*

**Figure 7:**
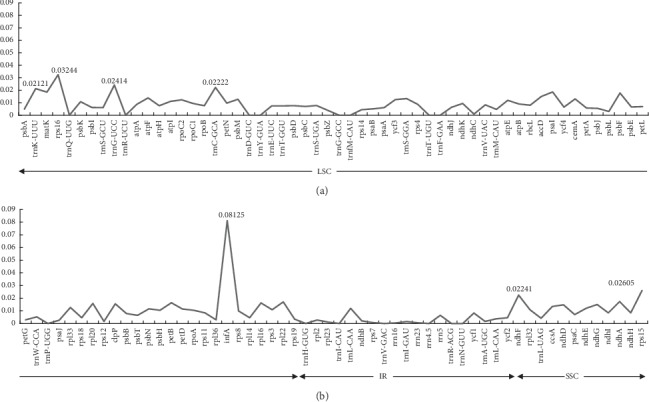
The nucleotide diversity of the shared 112 genes of the six species in chloroplast genomes.

We totally extracted the shared 112 genes of the six species in chloroplast genomes; the nucleotide variability (Pi) ranged from 0.00041 (*rrn16*) to 0.08125 (*infA*) among these shared genes (Figure 7; Table S2). Seven genes (*infA*, *rps16*, *rps15*, *ndhF*, *trnG-UCC*, *trnC-GCA*, and *trnK-UUU*) were considered to be hotspot regions with a nucleotide diversity greater than 0.02. These regions can be used to develop useful markers for phylogenetic analysis and distinguish the species in *Allium*.

### 3.6. Synonymous (Ks) and Nonsynonymous (Ka) Substitution Rate Analysis

**Figure 8:**
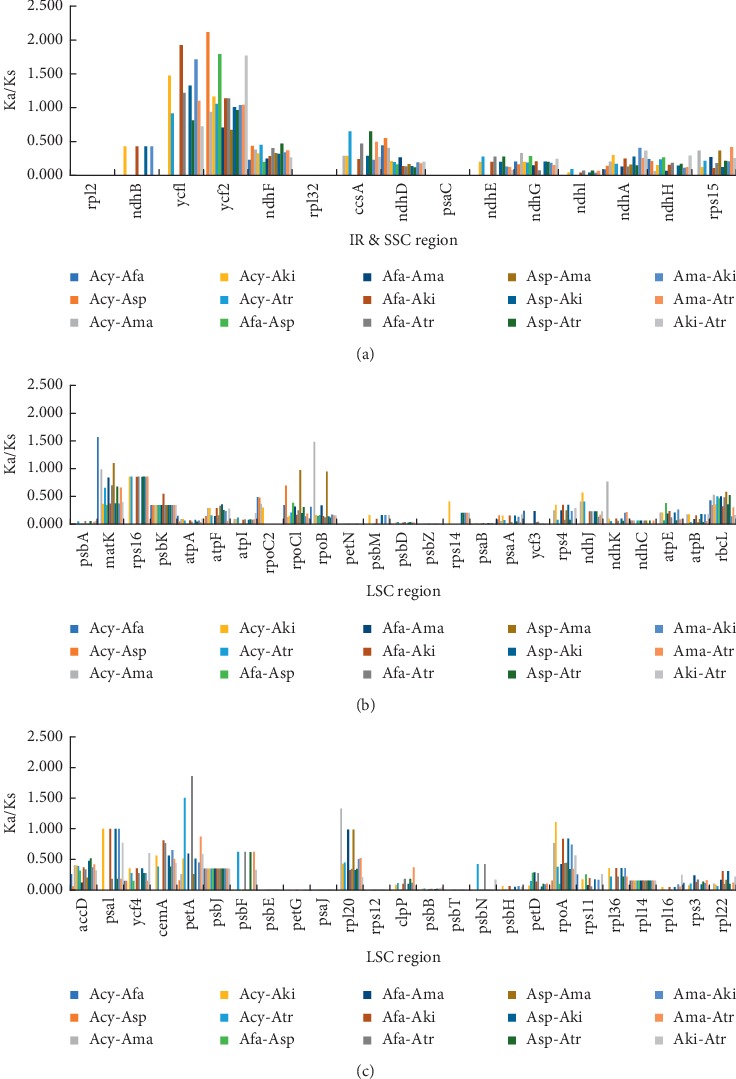
KA/KS analysis of 65 common protein coding genes in six *Allium* species. Acy, Afa Asp, Ama, Aki, and Atr stand for *A. cyathophorum*, *A. cyathophorum* var. *farreri*, *A. spicatum*, *A. mairei*, *A. kingdonii*, and *A. trifurcatum*, respectively. KA: nonsynonymous; KS: synonymous.

The Ka/Ks ratio is a significant index for understanding the evolution of protein-coding genes to assess gene differentiation rates and to determine whether positive, purified, or neutral selections have been performed; a Ka/Ks ratio >1 illustrates positive selection and Ka/Ks < 1 illustrates purifying selection, while the ratio of Ka/Ks close to 1 illustrates neutral selection [63]. In our study, the Ka/Ks ratio was calculated for 65 shared protein-coding genes in all six chloroplast genomes (Table S3), and the results are shown in Figure 8. The conservative genes with Ka/Ks ratio of 0.01, indicating powerful purifying selection pressure, were *rpl2*, *rpl32*, *psaC*, *psbA*, *rpoC2*, *petN*, *psbZ*, *psaB*, *psaJ*, and *psbT*, when the averaging Ka/Ks method showed *ycf1* and *ycf2* genes with Ka/Ks > 1, which shows that they may undergo some selective pressure among the six *Allium* species. The Ka/Ks ratios ranging from 0.5 to 1 were found for *matK*, *rps16*, *psaI*, *cemA*, *petA*, and *rpl20*, representing relaxed selection. The majority (56 of 65 genes) had an average Ka/Ks ratio ranging from 0 to 0.49 for the six compared groups, indicating that most genes were under purifying selection. Other than this, four genes (*matK*, *rpoB*, *petA*, and *rpoA*) with Ka/Ks > 1 in one or more pairwise comparisons (Figure 8) suggest that these genes may undergo selective pressure which is unknown, which is very important for researching the evolution of species.

### 3.7. Phylogenetic Analysis of Subgenus Cyathophora Depends on Chloroplast Genome

**Figure 9:**
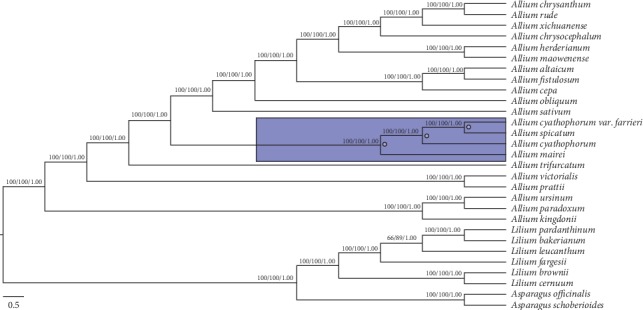
Phylogenetic relationship of subgenus *Cyathophora* with relational species. The whole chloroplast genomes dataset was analyzed using three different methods: maximum likelihood (ML), maximum parsimony (MP), and Bayesian inference (BI). Numbers on the branches stand for bootstrap values in the ML, MP, and posterior probabilities in the BI trees. Six species of *Lilium* and two species of *Asparagus* were considered as the out groups. Purple shows the branch of the subgenus *Cyathophora*.

The cp genome of sequence is significant and helpful to construct phylogenetic relationships and explore the evolutionary history in many previous reports [64, 65]. To explore the phylogenetic relationship of the six *Allium* species, we constructed the phylogenetic tree using three different methods and databases containing twenty-one *Allium* species, six *Lilium* species, and two *Asparagus* species as the out groups (Figure 9). Three databases of the complete genome sequences, the IGS sequences, and all CDS sequences using MP, BI, and ML methods all showed the same topologies with high support (Figures S2 and S3). The results strongly supported that subgenus *Cyathophora* is a monophyletic group, comprising *A. cyathophorum*, *A. cyathophorum* var. *farreri*, *A. spicatum*, and *A. mairei* in this study with 100% bootstrap value; subgenus *Cyathophora* does not contain *A. kingdonii* and *A. trifurcatum*, and the phylogenetic tree indicates that *A. cyathophorum* var. *farreri* is a direct sister to *A. spicatum*, which is in accordance with the results of previous molecular research studies [4, 6]. The sister relationship of *A. cyathophorum* var. *farreri* and *A. spicatum* strongly suggests that *A. spicatum* is closely related to subgenus *Cyathophora* though it is a special species with the significant abnormal spicate inflorescence compared to other species with capitate or umbellate inflorescence. Furthermore, *Allium kingdonii* was the closest relative of *Allium paradoxum* and *Allium ursinum*.

## 4. Discussion

### 4.1. Variations among the Six *Allium* Species

In this research, we assembled the complete cp genome of the six species in *Allium*. They were very conservative in genome structure and size; it showed a typical circular DNA structure and similar cp genome sequence length, ranging from 152,913 bp in *A. mairei* to 154,174 bp in *A. cyathophorum*. The six species had the identical numbers of protein-coding, tRNA, and rRNA genes. There were some expansion or contraction of IRs among these species (Figure 6); the expansion and contraction of IR regions are related to the divergences in chloroplast genome size [66]. To some extent, it is contributed to the cp genome variation and evolution. Other than this, variations in the IR/SC boundaries in the six cp genomes lead to the distinction in the whole length of sequence [61]. Previous research studies showed that SSRs have been widely known as important resources of molecular markers and have been broadly applied in phylogenetic and biogeographic studies [67, 68]. We surveyed and analyzed the quantities and distributions of SSRs with the six species in *Allium*, the largest number of SSR type was mononucleotide repeats, and the SSRs in the LSC area are much higher than those in the SSC and IR areas (Figure 4), showing that SSRs have a unevenly distribution in cp genome [50]. Additionally, we also explored seven common genes (*infA*, *rps16*, *rps15*, *ndhF*, *trnG-UCC*, *trnC-GCA*, and *trnK-UUU*) with nucleotide diversity more than 0.02 in the six cp genome sequences of *Allium*; among them, *trnK-UUU*, *trnG-UCC*, *ndhF*, and *rps15* have been previously known as hypervariable regions in *Allium* [17], and we consider that these SSRs and genes with greater nucleotide diversity can be used as helpful DNA barcodes to identify the species in *Allium*.

### 4.2. Phylogenetic Relationships

The results of phylogenetic analysis clearly show that *Allium* subgenus *Cyathophora* is a monophyletic group, and comprise four species (*A. cyathophorum*, *A. farreri*, *A. spicatum* and *A. mairei*), *A. cyathophorum* var. *farreri* has been upgraded to the level of the species as *A. farreri* in a recent study [69]. Besides *A. farreri* is a direct sister to *A. spicatum* with 100% strong bootstrap value, while the previous study showed low bootstrap value by the combined plastid dataset (t*rnL-F* + *rpl32-trnL*) [4]. Currently, most phylogenetic relationships are obtained with chloroplast fragments, while single ITS, chloroplast fragment, or chloroplast combined fragment does not have a better effect in phylogenetic analysis compared to the whole cp genome. We convinced that the complete chloroplast genomes have more advantages to solve the phylogenetic issues about the subgenus *Cyathophora*. In previous studies, many phylogenetic problems in many plants have been successfully resolved by using complete cp genome sequences [18, 19, 70]; the lately published article about *Allium* also well resolved the phylogenetic relationship [17, 71]. Although the morphological characteristics of the *A. farreri* and *A. spicatum* are obviously different, in which *A. spicatum* has distinctive spicate inflorescence compared to *A. farreri* with umbel hemispheric inflorescence, our results undoubtedly showed *A. farreri* is a direct sister to *A. spicatum*, which is in accordance with Li et al. [6]. According to previous study, different inflorescence may imply that the umbel inflorescence was replaced by spicate inflorescence to adapt the harsh environment [6]. The phylogenetic tree revealed *A. cyathophorum* had a closer relationship with *A. spicatum* and *A. farreri* compared to *A. mairei*. Furthermore, the members of subgenus *Cyathophora* do not contain *A. kingdonii* and *A. trifurcatum*; *A. kingdonii* was the closest relative of *A. paradoxum* and *A. ursinum*, which is consistence with previous studies [4, 6]. Certainly, our study persuasively constructed reliable phylogeny relationship of subgenus *Cyathophora* by using the complete cp genome data.

### 4.3. Selection Events in Protein Coding Genes

DNA base mutations can be divided into two categories based on their effects to the encoded amino acids: synonymous mutations (Ks) and nonsynonymous mutations (Ka). Synonymous mutations do not result in amino acid changes, the frequency of which is represented by Ks; nonsynonymous mutations resulted in a change of amino acid, the frequency of which is indicated by Ka [72]. The ratio (Ka/Ks) is an important indicator to reveal evolutionary rate and natural selection pressure [73]. Interestingly, the synonymous nucleotide substitutions occurred at a higher frequency than nonsynonymous substitutions, and thus Ka/Ks ratios are constantly <1 in most genes [9, 74], and our study is similar with this. Between different regions and genes, the Ka/Ks ratios were usually specific (Figure 8). Most conserved genes (56 of 65 genes) had an average Ka/Ks value ranging from 0 to 0.49 for the fifteen comparison groups, indicating that most genes were under purifying selection. On the contrary, the average Ka/Ks values of the *ycf1* and *ycf2* genes were >1 in the fifteen comparison groups, revealing that some selective pressure may execute on them in six *Allium* species. Previous studies have shown that *ycf1* and *ycf2* genes were two large open reading frames; they were important to tobacco, and the gene knockout experiments showed that *ycf1* and *ycf2* played important role in a healthy cell [75]. Hu et al. [76] suggested that plants have a variety of adaptation strategies in response to unforeseen environmental conditions. Recent studies about *Allium* species also suggested that the selective pressure in chloroplast genomes play an important role in *Allium* species adaptation and evolution [17, 71]. In our field investigations, the species of subgenus *Cyathophora* grows in slopes or grasslands with altitude ranging from 2700 m to 4800 m. The elevated Ka/Ks ratios observed about some genes in the six *Allium* species may suggest that it is relate to their specific living environment. What is more, there were four genes (*matK*, *rpoB*, *petA*, and *rpoA*) with Ka/Ks > 1 in at one or more pairwise comparisons (Figure 8, Table S3), and among these genes, *rpoA* was also undergone positive selection in species of Annonaceae [77]. Previous study demonstrated that *rpoA* encodes the *α* subunit of plastid RNA polymerase (PEP), which is in charge of the expression of most photosynthesis-related genes [78]. It is generally believed that low temperature and strong ultraviolet radiation are not conducive to effective photosynthesis of plants; therefore, plants that survive and reproduce at high altitudes need a special photosynthetic protection strategy [79, 80]. In this study, the population of subgenus *Cyathophora* is mainly distributed in the Qinghai-Tibet Plateau and its adjacent high-altitude regions [2]. Therefore, we speculated that the positive selection of these genes may be related to the difference between their optimal growth environment.

## 5. Conclusions

Here, we sequenced, assembled, and annotated six chloroplast genomes of *Allium* with high-throughput sequencing technology. The gene contents and orders of the cp genomes were extremely conservative, and their cp genomes are also quadripartite structure. Repeated sequence and SSRs are helpful sources for developing new molecular markers. Codon usage analyses detected that some amino acids of the six species showed distinct codon usage preferences, and we should comprehend codon usage bias to learn evolution process. We also discovered seven highly variable common genes which can be used to develop useful markers for phylogenetic analysis and distinguish species in *Allium*. The Ka/Ks analysis indicated that some selective pressure may exert on several genes in the chloroplast genomes of six *Allium* L. species. The maximum likelihood (ML), BI, and MP phylogenetic results clearly showed that subgenus *Cyathophora* comprised the four assembled species: *A. cyathophorum*, *A. cyathophorum* var. *farreri*, *A. spicatum*, and *A. mairei*, and *A. cyathophorum* var. *farreri* has a closer relationship with *A. spicatum*. This study will not only provide insights into the cp genome characteristics of species in subgenus *Cyathophora* but also supply useful genetic resources for phylogenetic analysis of genus *Allium*.

## Figures and Tables

**Table 1:** Samples information.

Species	Location	Geographical coordinate
*A. cyathophorum*	Mangkang, Tibet	29°43′24″N, 98°31′51″E
*A. cyathophorum* var. *farreri*	Zhouqu, Gansu	33°47′10″N, 104°22′7″E
*A. spicatum*	NaiDong, Tibet	29°13′40″N, 91°45′36″E
*A. mairei*	Shangri-La, Yunnan	27°25′19″N, 100°09′22″E
*A. trifurcatum*	Shangri-La, Yunnan	27°52′22″N, 99°43′10″E
*A. kingdonii*	Nyingchi, Tibet	29°37′27″N, 94°39′2″E

**Table 2:** Details comparison of the complete chloroplast genomes of six species of *Allium*.

	*A. cyathophorum*	*A. cyathophorum* var. *farreri*	*A. spicatum*	*A. mairei*	*A. trifurcatum*	*A. kingdonii*
Genome size (bp)	154,174	153,111	153,187	152,913	153,456	153,559
LSC length (bp)	83,359	82,544	82,625	82,493	82,628	83,423
SSC length (bp)	17,881	17,811	17,920	18,762	21,706	17,810
IR length (bp)	26,467	26,378	26,321	25,829	24,561	26,163
LSC GC content (%)	34.6	34.7	34.7	34.8	34.8	34.8
SSC GC content (%)	29.5	29.7	29.7	29.9	31.2	29.9
IR GC content (%)	42.7	42.7	42.7	42.9	43.1	42.7
Total GC content (%)	36.8	36.9	36.9	36.9	36.9	36.9
Total number of genes	132	132	132	132	132	132
Protein coding genes	86	86	86	86	86	86
rRNA	8	8	8	8	8	8
tRNA	38	38	38	38	38	38

**Table 3:** Genes existing in the chloroplast genome of *Allium*.

Category	Gene name	Number
Photosystem I	*psaA*, *psaB*, *psaC*, *psaI*, *psaJ*	5
Photosystem II	*psbA*, *psbB*, *psbC*, *psbD*, *psbE*, *psbF*, *psbH*, *psbI*, *psbJ*, *psbK*, *psbL*, *psbM*, *psbN*, *psbT*, *psbZ*	15
Cytochrome b6/f	*petA*, *petB*, *petD*, *petG*, *petL*, *petN*	6
ATP synthase	*atpA*, *atpB*, *atpE*, *atpF*, *atpH*, *atpI*	6
Rubisco	*rbcL*	1
NADH oxido reductase	*ndhA*, *ndhB*(×2), *ndhC*, *ndhD*, *ndhE*, *ndhF*, *ndhG*, *ndhH*, *ndhI*, *ndhJ*, *ndhK*	12
Large subunit ribosomal proteins	*rpl2*(×2), *rpl14*, *rpl16*, *rpl20*, *rpl22*, *rpl23*(×2), *rpl32*, *rpl33*, *rpl36*	11
Small subunit ribosomal proteins	rps*3*, rps*4*, rps*7*(×2), *rps8*, *rps11*, *rps12*(×2), *rps14*, *rps15*, *rps16*, *rps18*, *rps19*(×2)	14
RNAP	*rpoA*, *rpoB*, rpo*C1*, *rpoC2*	4
Other proteins	*accD*, *ccsA*, *matK*, *cemA*, *clpP*, *infA*	6
Proteins of unknown function	*ycf1*(×2), *ycf2*(×2), *ycf3*, *ycf4*	6
Ribosomal RNAs	*rrn23*(×2), *rrn16*(×2), *rrn5*(×2), *rrn4*.*5*(×2)	8
Transfer RNA	*trnA-UGC*(×2), *trnC-GCA*, *trnD-GUC*, *trnE-UUC*, *trnF-GAA*, *trnfM-CAU*, *trnH-GUG*(×2), *trnG-GCC*, *trnG-UCC*, *trnI-CAU*(×2), *trnI-GAU*(×2), *trnK-UUU*, *trnL-CAA*(×2), *trnL-UAA*, *trnL-UAG*, *trnM-CAU*, *trnN-GUU*(×2), *trnP-UGG*, *trnQ-UUG*, *trnR-ACG*(×2), *trnR-UCU*, *trnS-GCU*, *trnS-GGA*, *trnS-UGA*, *trnT-GGU*, *trnT-UGU*, *trnV-GAC*(×2), *trnV-UAC*, *trnW-CCA*, *trnY-GUA*	38
Total		132

**Table 4:** Codon usage in six *Allium* chloroplast genomes.

AA	Codon	Number						RSCU					
		Acy	Afa	Asp	Ama	Aki	Atr	Acy	Afa	Asp	Ama	Aki	Atr
Phe	UUU	826	825	827	822	809	827	**1.33**	**1.33**	**1.33**	**1.33**	**1.32**	**1.33**
	UUC	420	415	418	414	421	419	0.67	0.67	0.67	0.67	0.68	0.67
Leu	UUA	758	758	756	751	744	746	**2.05**	**2.05**	**2.05**	**2.05**	**2.04**	**2.04**
	UUG	447	450	447	441	446	444	**1.21**	**1.22**	**1.21**	**1.21**	**1.22**	**1.21**
	CUU	455	453	453	448	448	452	**1.23**	**1.23**	**1.23**	**1.22**	**1.23**	**1.24**
	CUC	138	137	138	139	136	138	0.37	0.37	0.37	0.38	0.37	0.38
	CUA	295	295	298	294	292	288	0.8	0.8	0.81	0.8	0.8	0.79
	CUG	122	121	124	122	119	125	0.33	0.33	0.34	0.33	0.33	0.34
Ile	AUU	943	946	945	955	938	931	**1.49**	**1.49**	**1.49**	**1.5**	**1.48**	**1.48**
	AUC	350	345	346	339	350	344	0.55	0.54	0.55	0.53	0.55	0.55
	AUA	611	610	613	615	616	616	0.96	0.96	0.97	0.97	0.97	0.98
Met	AUG	497	500	498	495	497	495	1	1	1	1	1	1
Val	GUU	431	432	432	429	440	438	**1.48**	**1.48**	**1.49**	**1.48**	**1.51**	**1.5**
	GUC	129	130	129	128	131	131	0.44	0.45	0.44	0.44	0.45	0.45
	GUA	432	435	434	434	430	433	**1.49**	**1.49**	**1.5**	**1.5**	**1.47**	**1.48**
	GUG	171	167	165	165	167	165	0.59	0.57	0.57	0.57	0.57	0.57
Ser	UCU	467	468	473	461	473	476	**1.73**	**1.73**	**1.75**	**1.72**	**1.76**	**1.76**
	UCC	246	254	249	252	249	244	0.91	0.94	0.92	0.94	0.93	0.9
	UCA	321	320	320	318	311	325	**1.19**	**1.18**	**1.18**	**1.19**	**1.16**	**1.2**
	UCG	151	151	150	151	159	154	0.56	0.56	0.55	0.56	0.59	0.57
Pro	CCU	338	339	338	337	338	338	**1.56**	**1.57**	**1.56**	**1.56**	**1.55**	**1.56**
	CCC	191	188	189	187	199	194	0.88	0.87	0.87	0.86	0.91	0.9
	CCA	247	244	247	243	246	238	**1.14**	**1.13**	**1.14**	**1.12**	**1.13**	**1.1**
	CCG	92	94	93	98	89	94	0.42	0.43	0.43	0.45	0.41	0.44
Thr	ACU	448	449	449	446	438	440	**1.65**	**1.67**	**1.67**	**1.65**	**1.63**	**1.63**
	ACC	186	184	184	185	191	193	0.69	0.68	0.68	0.69	0.71	0.72
	ACA	335	331	331	334	335	329	**1.24**	**1.23**	**1.23**	**1.24**	**1.25**	**1.22**
	ACG	116	114	114	114	111	115	0.43	0.42	0.42	0.42	0.41	0.43
Ala	GCU	533	536	538	537	528	542	**1.85**	**1.85**	**1.85**	**1.85**	**1.85**	**1.86**
	GCC	159	163	161	162	161	164	0.55	0.56	0.55	0.56	0.56	0.56
	GCA	343	345	348	341	335	348	**1.19**	**1.19**	**1.2**	**1.18**	**1.18**	**1.19**
	GCG	117	118	115	119	116	111	0.41	0.41	0.4	0.41	0.41	0.38
Tyr	UAU	683	669	668	674	679	666	**1.63**	**1.61**	**1.62**	**1.62**	**1.64**	**1.61**
	UAC	155	160	156	157	150	160	0.37	0.39	0.38	0.38	0.36	0.39
TER	UAA	29	29	29	29	30	26	**1.47**	**1.47**	**1.5**	**1.47**	**1.73**	**1.47**
	UAG	18	18	17	18	12	15	0.92	0.92	0.88	0.92	0.69	0.85
His	CAU	415	416	415	412	419	420	**1.56**	**1.57**	**1.56**	**1.57**	**1.56**	**1.56**
	CAC	118	113	116	113	118	119	0.44	0.43	0.44	0.43	0.44	0.44
Gln	CAA	583	585	585	587	587	591	**1.53**	**1.54**	**1.54**	**1.54**	**1.54**	**1.54**
	CAG	178	176	177	177	175	177	0.47	0.46	0.46	0.46	0.46	0.46
Asn	AAU	803	799	797	803	798	784	**1.56**	**1.56**	**1.56**	**1.56**	**1.55**	**1.55**
	AAC	229	226	227	226	231	227	0.44	0.44	0.44	0.44	0.45	0.45
Lys	AAA	876	878	884	866	838	863	**1.55**	**1.55**	**1.55**	**1.53**	**1.51**	**1.52**
	AAG	255	258	254	264	269	269	0.45	0.45	0.45	0.47	0.49	0.48
Asp	GAU	692	692	691	681	682	691	**1.64**	**1.64**	**1.64**	**1.63**	**1.62**	**1.62**
	GAC	154	152	154	156	158	160	0.36	0.36	0.36	0.37	0.38	0.38
Glu	GAA	861	864	857	861	869	852	**1.49**	**1.49**	**1.48**	**1.49**	**1.5**	**1.49**
	GAG	296	299	299	291	291	289	0.51	0.51	0.52	0.51	0.5	0.51
Cys	UGU	187	185	185	186	184	186	**1.52**	**1.54**	**1.54**	**1.54**	**1.52**	**1.54**
	UGC	59	56	56	55	58	56	0.48	0.46	0.46	0.46	0.48	0.46
TER	UGA	12	12	12	12	10	12	0.61	0.61	0.62	0.61	0.58	0.68
Trp	UGG	386	389	391	392	391	391	1	1	1	1	1	1
Arg	CGU	279	280	280	280	281	278	**1.37**	**1.37**	**1.37**	**1.38**	**1.37**	**1.35**
	CGC	74	78	78	76	71	80	0.36	0.38	0.38	0.37	0.34	0.39
	CGA	269	270	269	269	272	274	**1.32**	**1.32**	**1.31**	**1.32**	**1.32**	**1.33**
	CGG	88	89	87	88	89	89	0.43	0.43	0.43	0.43	0.43	0.43
Ser	AGU	339	342	342	342	331	335	**1.26**	**1.26**	**1.27**	**1.27**	**1.23**	**1.24**
	AGC	91	88	88	86	87	92	0.34	0.33	0.33	0.32	0.32	0.34
Arg	AGA	390	392	395	388	398	400	**1.91**	**1.91**	**1.93**	**1.91**	**1.93**	**1.94**
	AGG	125	121	119	119	124	119	0.61	0.59	0.58	0.59	0.6	0.58
Gly	GGU	489	487	487	493	483	489	**1.33**	**1.33**	**1.33**	**1.34**	**1.31**	**1.33**
	GGC	133	130	131	131	137	132	0.36	0.36	0.36	0.36	0.37	0.36
	GGA	620	621	625	622	623	616	**1.69**	**1.7**	**1.7**	**1.69**	**1.69**	**1.67**
	GGG	228	226	226	225	229	236	0.62	0.62	0.62	0.61	0.62	0.64

RSCU represents relative synonymous codon usage. RSCU more than one is highlighted in bold. Acy, Afa, Asp, Ama, Aki, and Atr stand for *A. cyathophorum*, *A. cyathophorum* var. *farreri*, *A. spicatum*, *A. mairei*, *A. kingdonii*, and *A. trifurcatum*, respectively.

## Data Availability

The complete chloroplast genome sequences of *A. cyathophorum*, *A. cyathophorum* var. *farreri*, *A. spicatum*, *A. mairei*, *Allium trifurcatum*, and *Allium kingdonii* are saved in the GenBank of NCBI, and the accession numbers are MK820611, MK931245, MK931246, MK820615, MK931247, and MK294559, respectively.

## Supplementary Materials

Supplementary 1 Table S1: all used accession numbers of cp genome sequences from GenBank in this article.

Supplementary 2 Table S2: the nucleotide variability (Pi) of 112 shared genes.

Supplementary 3 Table S3: the Ka/Ks ratio of 65 shared protein-coding genes in all six chloroplast genomes.

Supplementary 4 Figure S1: the proportion of the different lengths of the four dispersed repeats.

Supplementary 5 Figure S2: phylogenetic tree of the subgenus *Cyathophora* built by the CDS sequences using maximum likelihood (ML), maximum parsimony (MP), and Bayesian inference (BI).

Supplementary 6 Figure S3: phylogenetic tree of the subgenus *Cyathophora* built by the IGS sequences using maximum likelihood (ML), maximum parsimony (MP), and Bayesian inference (BI).

## References

[1] Friesen N., Fritsch R., Blattner F. (2006). Phylogeny and new intrageneric classification of allium (alliaceae) based on nuclear ribosomal DNA ITS sequences. *Aliso*.

[2] Li Q.-Q., Zhou S.-D., He X.-J., Yu Y., Zhang Y.-C., Wei X.-Q. (2010). Phylogeny and biogeography of allium (amaryllidaceae: allieae) based on nuclear ribosomal internal transcribed spacer and chloroplast rps16 sequences, focusing on the inclusion of species endemic to China. *Annals of Botany*.

[3] Friesen N., Fritsch R. M., Pollner S., Blattner F. R. (2000). Molecular and morphological evidence for an origin of the aberrant genus Milula within himalayan species of Allium (Alliacae). *Molecular Phylogenetics and Evolution*.

[4] Huang D.-Q., Yang J.-T., Zhou C.-J., Zhou S.-D., He X.-J. (2014). Phylogenetic reappraisal of allium subgenus cyathophora (amaryllidaceae) and related taxa, with a proposal of two new sections. *Journal of Plant Research*.

[5] Li M.-J., Guo X.-L., Li J., Zhou S.-D., Liu Q., He X.-J. (2017). Cytotaxonomy of allium (amaryllidaceae) subgenera cyathophora and amerallium sect. bromatorrhiza. *Phytotaxa*.

[6] Li M.-J., Tan J.-B., Xie D.-F., Huang D.-Q., Gao Y.-D., He X.-J. (2016). Revisiting the evolutionary events in Allium subgenus Cyathophora (amaryllidaceae): insights into the effect of the Hengduan mountains region (HMR) uplift and quaternary climatic fluctuations to the environmental changes in the Qinghai-Tibet Plateau. *Molecular Phylogenetics and Evolution*.

[7] Li B. (1987). On the boundaries of the Hengduan mountains. *Journal of Mountain Research*.

[8] Diels L. (1912). Plantae chinenses forrestianae: new and imperfectly known species. *Notes of the Royal Botanic Gardens Edinburgh*.

[9] Yin K., Zhang Y., Li Y., Du F. (2018). Different natural selection pressures on the atpF gene in evergreen sclerophyllous and deciduous oak species: evidence from comparative analysis of the complete chloroplast genome of quercus aquifolioides with other oak species. *International Journal of Molecular Sciences*.

[10] Olejniczak S. A., Łojewska E., Kowalczyk T., Sakowicz T. (2016). Chloroplasts: state of research and practical applications of plastome sequencing. *Planta*.

[11] Zhu A., Guo W., Gupta S., Fan W., Mower J. P. (2016). Evolutionary dynamics of the plastid inverted repeat: the effects of expansion, contraction, and loss on substitution rates. *New Phytologist*.

[12] Green B. R. (2011). Chloroplast genomes of photosynthetic eukaryotes. *The Plant Journal*.

[13] Jansen R. K., Raubeson L. A., Boore J. L. (2005). Methods for obtaining and analyzing whole chloroplast genome sequences. *Methods in Enzymology*.

[14] Burke S. V., Grennan C. P., Duvall M. R. (2012). Plastome sequences of two new world bamboos-Arundinaria gigantea and Cryptochloa strictiflora (poaceae)-extend phylogenomic understanding of bambusoideae. *American Journal of Botany*.

[15] Ma P.-F., Zhang Y.-X., Zeng C.-X., Guo Z.-H., Li D.-Z. (2014). Chloroplast phylogenomic analyses resolve deep-level relationships of an intractable bamboo tribe arundinarieae (poaceae). *Systematic Biology*.

[16] Xi Z., Ruhfel B. R., Schaefer H. (2012). Phylogenomics and a posteriori data partitioning resolve the Cretaceous angiosperm radiation Malpighiales. *Proceedings of the National Academy of Sciences*.

[17] Xie D. F., Yu H. X., Price M. (2019). Phylogeny of Chinese allium species in section daghestanica and adaptive evolution of allium (amaryllidaceae, allioideae) species revealed by the chloroplast complete genome. *Frontiers in Plant Science*.

[18] Yang Y., Zhu J., Feng L. (2018). Plastid genome comparative and phylogenetic analyses of the key genera in fagaceae: highlighting the effect of codon composition bias in phylogenetic inference. *Frontiers in Plant Science*.

[19] Kong H., Liu W., Yao G., Gong W. (2017). A comparison of chloroplast genome sequences in aconitum (Ranunculaceae): a traditional herbal medicinal genus. *PeerJ*.

[20] Gitzendanner M. A., Soltis P. S., Wong G. K.-S., Ruhfel B. R., Soltis D. E. (2018). Plastid phylogenomic analysis of green plants: a billion years of evolutionary history. *American Journal of Botany*.

[21] Scott-Phillips T. C., Laland K. N., Shuker D. M., Dickins T. E., West S. A. (2014). The niche construction perspective: a critical appraisal. *Evolution*.

[22] Yan C., Du J., Gao L., Li Y., Hou X. (2019). The complete chloroplast genome sequence of watercress (Nasturtium officinale R. Br.): genome organization, adaptive evolution and phylogenetic relationships in cardamineae. *Gene*.

[23] Altınordu F., Peruzzi L., Yu Y., He X. (2016). A tool for the analysis of chromosomes: KaryoType. *Taxon*.

[24] Andrews S., FastQC A. (2010). A quality control tool for high throughput sequence data. http://www.bioinformatics.babraham.ac.uk/projects/fastqc.

[25] Luo R., Liu B., Xie Y. (2012). SOAPdenovo2: an empirically improved memory-efficient short-read de novo assembler. *Gigascience*.

[26] Dierckxsens N., Mardulyn P., Smits G. (2016). NOVOPlasty: de novo assembly of organelle genomes from whole genome data. *Nucleic Acids Research*.

[27] Kearse M., Moir R., Wilson A. (2012). Geneious Basic: an integrated and extendable desktop software platform for the organization and analysis of sequence data. *Bioinformatics*.

[28] Lohse M., Drechsel O., Kahlau S., Bock R. (2013). OrganellarGenomeDRAW-a suite of tools for generating physical maps of plastid and mitochondrial genomes and visualizing expression data sets. *Nucleic Acids Research*.

[29] Kurtz S., Choudhuri J. V., Ohlebusch E., Schleiermacher C., Stoye J., Giegerich R. (2001). REPuter: the manifold applications of repeat analysis on a genomic scale. *Nucleic Acids Research*.

[30] Mudunuri S. B., Nagarajaram H. A. (2007). IMEx: imperfect microsatellite extractor. *Bioinformatics*.

[31] Peden J. (1997). *CodonW*.

[32] Wright F. (1990). The “effective number of codons” used in a gene. *Gene*.

[33] Frazer K. A., Pachter L., Poliakov A., Rubin E. M., Dubchak I. (2004). VISTA: computational tools for comparative genomics. *Nucleic Acids Research*.

[34] Librado P., Rozas J. (2009). DnaSP v5: a software for comprehensive analysis of DNA polymorphism data. *Bioinformatics*.

[35] Wang D., Zhang Y., Zhang Z., Zhu J., Yu J. (2010). KaKs_Calculator 2.0: a toolkit incorporating gamma-series methods and sliding window strategies. *Genomics, Proteomics & Bioinformatics*.

[36] Katoh K., Standley D. M. (2013). MAFFT multiple sequence alignment software version 7: improvements in performance and usability. *Molecular Biology and Evolution*.

[37] Posada D., Crandall K. A. (2001). Selecting the best-fit model of nucleotide substitution. *Systematic Biology*.

[38] Stamatakis A. (2006). RAxML-VI-HPC: maximum likelihood-based phylogenetic analyses with thousands of taxa and mixed models. *Bioinformatics*.

[39] Swofford D. L. (2002). *Phylogenetic Analysis Using Parsimony (∗ and Other Methods)*.

[40] Ronquist F., Teslenko M., van der Mark P. (2012). MrBayes 3.2: efficient Bayesian phylogenetic inference and model choice across a large model space. *Systematic Biology*.

[41] Lee J., Chon J., Lim J., Kim E.-K., Nah G. (2017). Characterization of complete chloroplast genome of Allium victorialis and its application for barcode markers. *Plant Breeding and Biotechnology*.

[42] Bull L. N., Pabon-Pena C. R., Freimer N. B. (1999). Compound microsatellite repeats: practical and theoretical features. *Genome Research*.

[43] Asano T., Tsudzuki T., Takahashi S., Shimada H., Kadowaki K.-i. (2004). Complete nucleotide sequence of the sugarcane (Saccharum officinarum) chloroplast genome: a comparative analysis of four monocot chloroplast genomes. *DNA Research*.

[44] Gao L., Yi X., Yang Y.-X., Su Y.-J., Wang T. (2009). Complete chloroplast genome sequence of a tree fern Alsophila spinulosa: insights into evolutionary changes in fern chloroplast genomes. *BMC Evolutionary Biology*.

[45] Chen C., Zheng Y., Liu S. (2017). The complete chloroplast genome of Cinnamomum camphora and its comparison with related Lauraceae species. *PeerJ*.

[46] Xie D.-F., Yu Y., Deng Y.-Q. (2018). Comparative analysis of the chloroplast genomes of the Chinese endemic genus Urophysa and their contribution to chloroplast phylogeny and adaptive evolution. *International Journal of Molecular Sciences*.

[47] Nie X., Lv S., Zhang Y. (2012). Complete chloroplast genome sequence of a major invasive species, crofton weed (Ageratina adenophora). *PLoS One*.

[48] Chen J., Hao Z., Xu H. (2015). The complete chloroplast genome sequence of the relict woody plant Metasequoia glyptostroboides Hu et Cheng. *Frontiers in Plant Science*.

[49] Kuang D.-Y., Wu H., Wang Y.-L., Gao L.-M., Zhang S.-Z., Lu L. (2011). Complete chloroplast genome sequence of Magnolia kwangsiensis (Magnoliaceae): implication for DNA barcoding and population genetics. *Genome*.

[50] Qian J., Song J., Gao H. (2013). The complete chloroplast genome sequence of the medicinal plant Salvia miltiorrhiza. *PLoS One*.

[51] Wong G. K.-S., Wang J., Tao L. (2002). Compositional gradients in Gramineae genes. *Genome Research*.

[52] Ermolaeva M. D. (2001). Synonymous codon usage in bacteria. *Current Issues in Molecular Biology*.

[53] Wang W., Yu H., Wang J. (2017). The complete chloroplast genome sequences of the medicinal plant forsythia suspensa (oleaceae). *International Journal of Molecular Sciences*.

[54] Yi D.-K., Kim K.-J. (2012). Complete chloroplast genome sequences of important oilseed crop Sesamum indicum L. *PloS One*.

[55] Yu X., Zuo L., Lu D., Lu B., Yang M., Wang J. (2019). Comparative analysis of chloroplast genomes of five Robinia species: genome comparative and evolution analysis. *Gene*.

[56] Dong W., Xu C., Cheng T., Zhou S. (2013). Complete chloroplast genome of Sedum sarmentosum and chloroplast genome evolution in Saxifragales. *PLoS One*.

[57] Zhang Y. J., Du L. W., Liu A. (2016). The complete chloroplast genome sequences of five epimedium species: lights into phylogenetic and taxonomic analyses. *Frontiers in Plant Science*.

[58] Liu L. X., Wang Y. W., He P. Z. (2018). Chloroplast genome analyses and genomic resource development for epilithic sister genera oresitrophe and mukdenia (saxifragaceae), using genome skimming data. *BMC Genomics*.

[59] Menezes A. P. A., Resende-Moreira L. C., Buzatti R. S. O. (2018). Chloroplast genomes of byrsonima species (malpighiaceae): comparative analysis and screening of high divergence sequences. *Scientific Reports*.

[60] Goulding S. E., Olmstead R. G., Morden C. W., Wolfe K. H. (1996). Ebb and flow of the chloroplast inverted repeat. *Molecular and General Genetics MGG*.

[61] Yang M., Zhang X., Liu G. (2010). The complete chloroplast genome sequence of date palm (Phoenix dactylifera L.). *PLoS One*.

[62] Wang R.-J., Cheng C.-L., Chang C.-C., Wu C.-L., Su T.-M., Chaw S.-M. (2008). Dynamics and evolution of the inverted repeat-large single copy junctions in the chloroplast genomes of monocots. *BMC Evolutionary Biology*.

[63] Kimura M. (1989). The neutral theory of molecular evolution and the world view of the neutralists. *Genome*.

[64] Hong S. Y., Cheon K. S., Yoo K. O. (2017). Complete chloroplast genome sequences and comparative analysis of Chenopodium quinoa and C. album. *Frontiers in Plant Science*.

[65] Chaney L., Mangelson R., Ramaraj T., Jellen E. N., Maughan P. J. (2016). The complete chloroplast genome sequences for four amaranthus species (amaranthaceae). *Applications in Plant Sciences*.

[66] Ravi V., Khurana J. P., Tyagi A. K., Khurana P. (2008). An update on chloroplast genomes. *Plant Systematics and Evolution*.

[67] Pauwels M., Vekemans X., Godé C., Frérot H., Castric V., Saumitou-Laprade P. (2012). Nuclear and chloroplast DNA phylogeography reveals vicariance among European populations of the model species for the study of metal tolerance, Arabidopsis halleri (brassicaceae). *New Phytologist*.

[68] Powell W., Morgante M., McDevitt R., Vendramin G. G., Rafalski J. A. (1995). Polymorphic simple sequence repeat regions in chloroplast genomes: applications to the population genetics of pines. *Proceedings of the National Academy of Sciences*.

[69] Li M. J., Liu J. Q., Guo X. L., Xiao Q. Y., He X. J. (2019). Taxonomic revision of Allium cyathophorum (amaryllidaceae). *Phytotaxa*.

[70] Jansen R. K., Cai Z., Raubeson L. A. (2007). Analysis of 81 genes from 64 plastid genomes resolves relationships in angiosperms and identifies genome-scale evolutionary patterns. *Proceedings of the National Academy of Sciences*.

[71] Huo Y., Gao L., Liu B. (2019). Complete chloroplast genome sequences of four Allium species: comparative and phylogenetic analyses. *Scientific Reports*.

[72] Hurst L. D. (2002). The Ka/Ks ratio: diagnosing the form of sequence evolution. *Trends Genetics*.

[73] Yang Z., Nielsen R. (2000). Estimating synonymous and nonsynonymous substitution rates under realistic evolutionary models. *Molecular Biology and Evolution*.

[74] Makałowski W., Boguski M. S. (1998). Evolutionary parameters of the transcribed mammalian genome: an analysis of 2,820 orthologous rodent and human sequences. *Proceedings of the National Academy of Sciences*.

[75] Drescher A., Ruf S., Calsa T., Carrer H., Bock R. (2000). The two largest chloroplast genome‐encoded open reading frames of higher plants are essential genes. *The Plant Journal*.

[76] Hu H., Al-Shehbaz I. A., Sun Y. S., Hao G. Q., Wang Q., Liu J. Q. (2015). Species delimitation in orychophragmus (brassicaceae) based on chloroplast and nuclear DNA barcodes. *Taxon*.

[77] Blazier J. C., Ruhlman T. A., Weng M.-L., Rehman S. K., Sabir J. S., Jansen R. K. (2016). Divergence of RNA polymerase *α* subunits in angiosperm plastid genomes is mediated by genomic rearrangement. *Scientific Reports*.

[78] Hajdukiewicz P. T., Allison L. A., Maliga P. (1997). The two RNA polymerases encoded by the nuclear and the plastid compartments transcribe distinct groups of genes in tobacco plastids. *The EMBO Journal*.

[79] Streb P., Aubert S., Gout E., Bligny R. (2003). Cold‐and light‐induced changes of metabolite and antioxidant levels in two high mountain plant species Soldanella alpina and Ranunculus glacialis and a lowland species Pisum sativum. *Physiologia Plantarum*.

[80] Ikeda H., Fujii N., Setoguchi H. (2009). Molecular evolution of phytochromes in Cardamine nipponica (brassicaceae) suggests the involvement of PHYE in local adaptation. *Genetics*.

