# Supplementary material for: Comparative Analysis of the Complete Chloroplast Genomes in Allium Subgenus Cyathophora (Amaryllidaceae): Phylogenetic Relationship and Adaptive Evolution
Source: Biomed Res Int. 2020 Jan 17;2020:1732586. doi: 10.1155/2020/1732586 (PMC7201574; doi:10.1155/2020/1732586)
Supplement: Supplementary 1 — Table S1: all used accession numbers of cp genome sequences from GenBank in this article. [file 1732586.f1.docx]

**Table S1** **all used accession numbers of cp genome sequences from GenBank in this article.**

| **Species** | **GenBank accession numbers** |
| --- | --- |
| *Allium cyathophorum* | MK820611 |
| *Allium cyathophorum var. farrieri* | MK931245 |
| *Allium spicatum* | MK931246 |
| *Allium mairei* | MK820615 |
| *Allium trifurcatum* | MK931247 |
| *Allium kingdonii* | MK294559 |
| *Allium fistulosum* | MH926357 |
| *Allium altaicum* | MH159130 |
| *Allium cepa* | KM088014 |
| *Allium maowenense* | MH992111 |
| *Allium herderianum* | MH992110 |
| *Allium xichuanense* | MH992113 |
| *Allium rude* | MH992112 |
| *Allium chrysanthum* | MH992108 |
| *Allium chrysocephalum* | MH992109 |
| *Allium obliquum* | NC 037199 |
| *Allium sativum* | KX683282 |
| *Allium victorialis* | NC 037240 |
| *Allium prattii* | NC 037432 |
| *Allium ursinum* | MH157875 |
| *Allium paradoxum* | MH053150 |
| *Lilium bakerianum* | NC 035592 |
| *Lilium pardanthinum* | MG704135 |
| *Lilium leucanthum* | NC035590 |
| *Lilium fargesii* | NC033908 |
| *Lilium brownii* | KY748296 |
| *Lilium cernuum* | NC034840 |
| *Asparagus officinalis* | NC 034777 |
| *Asparagus schoberioides* | NC 035969 |
