# Supplementary figures and images for: Comparative Analysis of the Complete Chloroplast Genomes in Allium Subgenus Cyathophora (Amaryllidaceae): Phylogenetic Relationship and Adaptive Evolution
Source: Biomed Res Int. 2020 Jan 17;2020:1732586. doi: 10.1155/2020/1732586 (PMC7201574; doi:10.1155/2020/1732586)

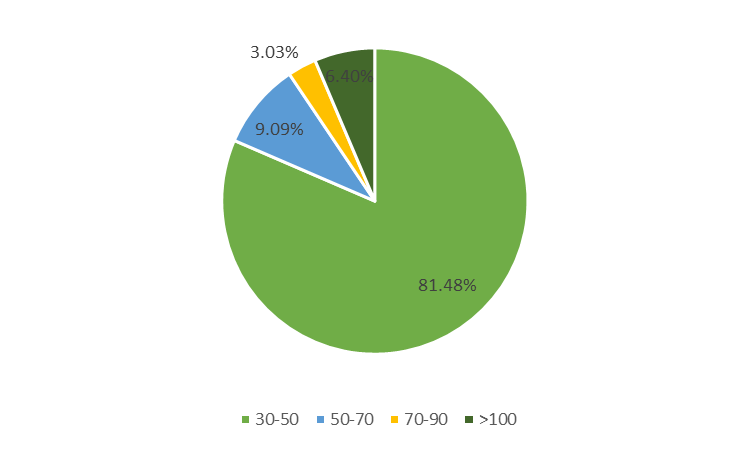


FigureS1. The proportion of the different lengths of the four dispersed repeats.

Supplement: Supplementary 4 — Figure S1: the proportion of the different lengths of the four dispersed repeats. [file 1732586.f4.docx]
