# Supplementary material for: Comparative Analysis of the Complete Chloroplast Genomes in Allium Subgenus Cyathophora (Amaryllidaceae): Phylogenetic Relationship and Adaptive Evolution
Source: Biomed Res Int. 2020 Jan 17;2020:1732586. doi: 10.1155/2020/1732586 (PMC7201574; doi:10.1155/2020/1732586)
Supplement: Supplementary 5 — Figure S2: phylogenetic tree of the subgenus Cyathophora built by the CDS sequences using maximum likelihood (ML), maximum parsimony (MP), and Bayesian inference (BI). [file 1732586.f5.docx]

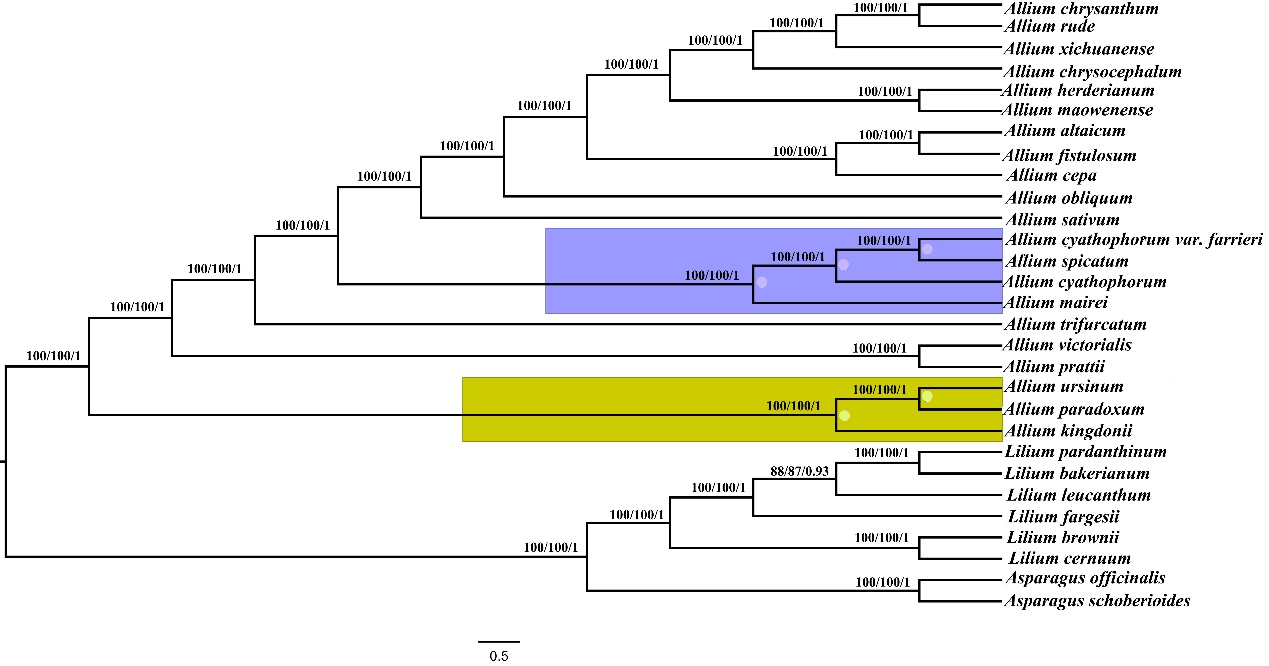


Figure S2. Phylogenetic tree of subgenus *Cyathophora* with related species. The CDS dataset was analyzed using three different methods: maximum likelihood (ML), maximum parsimony (MP), and Bayesian inference (BI). Numbers above the branches represent bootstrap values in the ML, MP, and posterior probabilities in the BI trees, whereas the number below the branches represents branch length.
